# Isolation, Characterization and Potential Role in Beta Cell-Endothelium Cross-Talk of Extracellular Vesicles Released from Human Pancreatic Islets

**DOI:** 10.1371/journal.pone.0102521

**Published:** 2014-07-16

**Authors:** Federico Figliolini, Vincenzo Cantaluppi, Michela De Lena, Silvia Beltramo, Renato Romagnoli, Mauro Salizzoni, Raffaella Melzi, Rita Nano, Lorenzo Piemonti, Ciro Tetta, Luigi Biancone, Giovanni Camussi

**Affiliations:** 1 Department of Medical Sciences, University of Torino, Torino, Italy; 2 Liver Transplantation Center, University of Torino, Torino, Italy; 3 Diabetes Research Institute (HSR-DRI), San Raffaele Scientific Institute, Milano, Italy; 4 Fresenius Medical Care, Bad Homburg, Germany; University of Lille Nord de France, France

## Abstract

The cross-talk between beta cells and endothelium plays a key role in islet physiopathology and in the revascularization process after islet transplantation. However, the molecular mechanisms involved in this cross-talk are not fully elucidated. Extracellular vesicles (EVs) are secreted membrane nanoparticles involved in inter-cellular communication through the transfer of proteins and nucleic acids. The aims of this study were: 1) isolation and characterization of EVs from human islets; 2) evaluation of the pro-angiogenic effect of islet-derived EVs on human islet endothelial cells (IECs). EVs were isolated by ultracentrifugation from conditioned medium of human islets and characterized by nanotrack analysis (Nanosight), FACS, western blot, bioanalyzer, mRNA/microRNA RT-PCR array. On IECs, we evaluated EV-induced insulin mRNA transfer, proliferation, resistance to apoptosis, *in vitro* angiogenesis, migration, gene and protein profiling. EVs sized 236±54 nm, expressed different surface molecules and islet-specific proteins (insulin, C-peptide, GLP1R) and carried several mRNAs (VEGFa, eNOS) and microRNAs (miR-27b, miR-126, miR-130 and miR-296) involved in beta cell function, insulin secretion and angiogenesis. Purified EVs were internalized into IECs inducing insulin mRNA expression, protection from apoptosis and enhancement of angiogenesis. Human islets release biologically active EVs able to shuttle specific mRNAs and microRNAs (miRNAs) into target endothelial cells. These results suggest a putative role for islet-derived EVs in beta cell-endothelium cross-talk and in the neoangiogenesis process which is critical for engraftment of transplanted islets.

## Introduction

The cross-talk between beta cells and endothelial cells represents a key mechanism in the physiopathology of pancreatic islets [1], [2]. Islet endothelial cells (IECs) release molecules such as hepatocyte growth factor involved in beta cell proliferation and differentiation [3]. On the other hand, beta cells secrete pro-angiogenic factors that are able to promote islet vascularization [4]–[6]. The maintenance of endothelial integrity is relevant not only in native islets but also in islet transplantation, where lack of revascularization is a main cause of loss of graft function [7], [8].

It has been demonstrated that conditioned medium derived from cultured rat islets induces liver and islet-derived endothelial cell proliferation and migration *in vitro* [9], suggesting the presence of paracrine pro-angiogenic factors. Extracellular vesicles (EVs) represent a newly discovered mechanism of cell-to-cell communication. EVs are released by a membrane sorting process from different types of activated cells influencing the behaviour of surrounding target cells through the transfer of bioactive lipids, proteins, receptors and genetic information [10]–[12]. We recently demonstrated that EVs derived from endothelial progenitor cells (EPCs) activate an angiogenic program in quiescent endothelial cells through the horizontal transfer of RNAs [13]. In addition, EPC-derived EVs enhance neo-angiogenesis in transplanted pancreatic islets through mRNA and microRNA transfer [14]. Other types of fully differentiated cells have been shown to produce biologically active EVs [15]–[17]. However, the release of EVs from human pancreatic islets has not been investigated yet. The aim of the present study was to isolate EVs released from human islets and to evaluate their potential role in beta cell-endothelium cross-talk and in islet angiogenesis.

## Materials and Methods

### Isolation and characterization of islets and islet-derived EVs

The use of human specimens (supernatant of cultured islets or islets discarded from clinical use) was approved by Institutional Review Board. Islet-free supernatants were obtained from freshly purified human islet preparations (n = 10) from Diabetes Research Institute (HSR-DRI), San Raffaele Scientific Institute, Milan, Italy. Islets were isolated and purified according to the automated method described by Ricordi, with local modifications [18]. Briefly, the pancreatic duct was cannulated with 14–20 G catheter and distended by intraductal infusion of a cold collagenase solution (Collagenase NB1 Premium Grade, Serva, Heidelberg, Germany). After digestion at 37°C in a modified Ricordi chamber, islets were purified on a Cobe 2991 (TerumoBCT, Lakewood, CO, USA) using a continuous HBSS-Ficoll (Biochrom, Berlin, Germany) gradient. Purified islet fractions were cultured in Final Wash (Mediatech Cellgro, VA, USA) plus 1% Pen/Strep, 1% Glutamine (Lonza, Basel, Switzerland), counted and their numbers expressed as number of islets normalized to a 150-µm diameter (IEQ). Final islet preparations were cultured at a density of 1000±150 IEQ/ml.

Human islet preparations were selected for purity (>70%) and viability (>70%) for EV isolation by Dithizone (Sigma-Aldrich, St. Louis, MO) and Fluorescein Diacetate/Propidium Iodide (FDA/PI; (Sigma-Aldrich, St. Louis, MO) dye analysis.

### Fluorescein Diacetate/Propidium Iodide (FDA/PI)

Supernatants (100 ml each) were collected for 24 hrs, submitted to 3 centrifugations at 4,000×g for 20 minutes (4 K fraction) to eliminate islet and cell debris. The islet-free supernatants were centrifuged at 100,000×g (Beckman Coulter Optima l-90K; Fullerton, CA) for 1 hr at 4°C. EVs were washed in serum-free medium 199 (Sigma-Aldrich) containing 25 mM HEPES and submitted to a second ultracentrifugation at 100,000×g for 1 hr at 4°C and resuspended in 200 µl of serum-free medium 199 (Sigma-Aldrich). The protein content of EVs was quantified by Bradford method (Biorad, Hercules, CA, USA) and EVs were stored at −80°C. In selected experiments, islet-free supernatants were subjected to serial centrifugation: the first one at 10,000×g (10 K fraction) and the second one at 100,000×g (100 K fraction) to evaluate the presence of apoptotic bodies and the biological activity of different EV fractions by BrdU-based assay (see below). To trace EVs for FACS analysis, after isolation EVs were labeled with red fluorescence aliphatic chromophore intercalating into lipid bilayers PKH26 (Sigma-Aldrich) or with DNA intercalating dye, that differentially stains double-stranded and single-stranded nucleic acids, acridine orange solution (Sigma-Aldrich). In selected experiments, EVs were treated with 1 U/ml RNase (Ambion Inc., Austin, TX) as previously described [13], [14]. The reaction was stopped by addition of 10 U/ml RNAse inhibitor (Ambion) and EVs were washed by ultracentrifugation.

### Nanosight analysis

EVs were analysed by Nanosight LM10 system (Nanosight Ltd., Amesbury, UK). Briefly, EV preparations were diluted (1∶100) in sterile saline solution 0.9% and analysed by NanoSight LM10 equipped with the Nanoparticle Analysis System & NTA 1.4 Analytical Software [14], [19].

### Isolation and characterization of IECs

Freshly purified human islet preparations (n = 5), discarded from transplant use for insufficient islet mass, were cultured, after approval by the local ethical committee, on tissue culture treated flasks in CMRL1066 with 10% FBS (Life Technologies, Carlsbad, CA). After 7 days of culture, cells derived from the outgrowth were transfected with pBR322 plasmid vector containing SV40-T large antigen gene and clones sorted for CD31 and characterized as previously described [20].

### GUAVA FACS analysis

FACS analysis was performed with GUAVA (GUAVA EasyCyte, Millipore) that allows direct detection of nanoparticles with about 200 nm [21], using APC-, PE- or FITC-conjugated antibodies directed to α4, α6, β1(CD29) integrins, HLA I, HLA II, CD44, CD31, ICAM-1 (Beckton Dickinson, Franklin Lakes, NJ), GLP1R and glucagon (Santa Cruz, Dallas, TX). FITC or PE mouse nonimmune isotypic IgG (Beckton Dickinson, Franklin Lakes, NJ) was used as control. In particular, EVs were incubated with each antibody, or isotype control antibody, with or without permeabilization solution, for 1 hour at 4°C and after appropriate washing and centrifugation, analysed by FACS. By cytofluorimetric analysis EVs were detected mainly below the forward scatter signal corresponding to 1 µm beads (data not shown).

To evaluate EV internalization, IECs were incubated with different doses of PKH26-labelled EVs (0, 5, 10, 25, 50, 100 µg/ml) for different time points (0, 3, 6, 12, 24 hours) in serum free medium and then, after appropriate washing, analysed by FACS.

In selected experiments, blocking antibodies or peptides directed to different integrins (α6, α4, β1(CD29) and ICAM1 (Millipore, Billerica, MA) or soluble hyaluronic acid (HA; Sigma, St. Louis, MO) were used. Briefly, EVs were pre-incubated for 1 hr at 4°C with 1 µg/ml of blocking antibodies or 5 µg/ml of soluble HA and then, after appropriate washing and centrifugation, EVs were incubated with IECs for 30 minutes in serum free medium. After incubation with EVs, IECs were washed and analyzed by FACS.

### RNA extraction and Bioanalyzer analysis

Total RNA from islets and from islet EVs was extracted by Mirvana kit (Ambion Inc, Austin, TX) according to manufacturer’s instructions. Analysis of RNAs contained in islets and in RNase-treated and untreated islet EVs was assessed by Agilent 2100 Bioanalyzer (Agilent Tech, Inc., Santa Clara, CA) using the eukaryotic total RNA 6000 Nano kit and small RNA kit (Agilent Tech.).

### DNA extraction and Agarose gel electrophoresis analysis

Total DNA from 10 k and 100 k fraction EVs was extracted by all-in-one purification kit (Norgen biotek corp., Thorold, Canada) according to manufacturer’s instructions. DNA contained in islet EVs was subjected to 1% agarose gel electrophoresis containing ethidium bromide and analysed under ultraviolet (UV) light.

### RT-PCR array analysis of islet EVs and IECs

Human diabetes (PAHS-023: European Bioinformatics Institute E-MEXP-3695; www.ebi.ac.uk) and human angiogenesis (PAHS-024: European Bioinformatics Institute E-MEXP-3693; www.ebi.ac.uk) RT^2^ profiler PCR arrays (SABiosciences, Frederick, MD, USA) were used to characterize respectively the gene expression profiles of human islet-derived EVs or IECs stimulated or not with 50 µg/ml of islet EVs for 12 and 24 hrs.

Briefly, according to manufacturer’s instructions, RNA extracted from islet EVs and IECs was treated with gDNA elimination buffer, to degrade DNA contamination, and then reverse-transcripted by using RT^2^ first strand kits. RT products were loaded into 96 well array and Real Time RT-PCR amplification was performed by using the Bio-Rad iCycler Real Time PCR System. Data analysis was performed using RT^2^ Profiler PCR Array Data Analysis tool provided by manufacturer (SABioscience) and the expression levels of each gene were normalized for housekeeping genes according to manufacturer’s instruction.

### MicroRNA analysis in islet EVs

MicroRNA (miRNA) analysis was performed by TaqMan Human MicroRNA A card (Life Technologies, Carlsbad, CA) in accordance to manufacturer’s instructions (European Bioinformatics Institute E-MEXP-3691; www.ebi.ac.uk). Briefly, RNAs isolated from islets and islet EVs were reverse-transcripted by using Megaplex RT kit containing RT primers, allowing reverse transcription of all 365 miRNA targets in one RT reaction. The RT reaction products was submitted to a preamplification reaction, consisting of 12 cycles reaction, to improve signal for small quantity of RNA as indicated in user’s guide. Finally, preamplification reaction products were simply loaded into one of the eight filling ports on the TaqMan Array and then Real Time RT-PCR Amplification was performed by using the Applied Biosystem 7900 HT Fast Real Time PCR System. Data Analysis was performed by a bioinformatic software (SDS manager) to analyze and find cycle amplification differences among miRNAs and to normalize all miRNAs data with snoRNA data (endogenous control) present in the same array in accordance to manufacturer’s instructions.

### Western blot analysis of islets, islet EVs and IECs

Islets, islet-derived EVs or IECs stimulated or not with 50 µg/ml of islet EVs or RNase pre-treated islet EVs at different time points were lysed at 4°C for 1 hour in a lysis buffer (50 mM Tris-HCl, pH8.3, containing 1% Triton X-100, 1 mM PMSF, 10 µg/mL leupeptin and 100 U/mL aprotinin) then quantified using Bradford method by Nanodrop 1000 (Nanodrop, Wilmington, DE, USA) and analyzed by western blot. Briefly, 30 µg of sample lysates were subjected to 4% to 15% gradient sodium dodecyl sulfate-polyacrylamide gel electrophoresis (SDS-PAGE) under reducing conditions and electroblotted onto nitrocellulose membrane filters. The following primary antibodies were used: human insulin, GLUT 2, PDX 1, AGO2, Angiopoietin-1, CD63, VEGF-A, VEGFR1, VEGFR2, AKT, p-AKT, eNOS, p-eNOS, ERK, p-ERK, BAD, Bcl-2, Thrombospondin-1, actin (Santa Cruz Biotech, Santa Cruz, CA) and human C-peptide (Abcam, Cambridge, MA). After incubation for 12 hours at 4°C with primary antibodies, nitrocellulose membrane filters were washed with PBS 10% BSA 0.5% tween 20 and then incubated with specific HRP-conjugated secondary antibodies for 1 hour at room temperature. After washing step, nitrocellulose membrane filters were incubated with ECL western blot detection system (GE healthcare, Amersham, Buckinghamshire, UK) for 10 minutes and images were captured by ChemiDoc XRS system (Biorad). Images of nitrocellulose membrane filters were analyzed by ImageJ software (Image processing and analysis in Jave) to quantify size and strength of protein bands.

### In vitro assays on IECs

IEC proliferation was studied by 5-bromo-2-deoxyuridine (BrdU) (Roche Diagnostics, Mannheim, Germany) incorporation in cells stimulated or not with different doses of islet EVs and with RNase pre-treated EVs (50 µg/ml) for 12 hrs.

Briefly, IECs were cultured on 24-well and stimulated or not with different doses of islet EVs (1, 5, 10, 25, 50 µg/ml) and with RNase pre-treated EVs (50 µg/ml) for 12 hours in RPMI without serum in presence of 10 µM BrdU (Roche Diagnostics, Mannheim, Germany). After incubation, cells were washed and fixed with fixing solution, treated with nucleases to digest cellular DNA and then incubated with anti-BrdU-POD antibody. Finally, peroxidase substrate ABTS was added and peroxidase catalysed the cleavage of ABTS producing a colored reaction product directly proportional to the amount of BrdU integrated into cell DNA. In addition, different fractions of EVs obtained by serial ultracentrifugation were also tested and in selected experiments, EVs (50 µg/ml) were pre-treated with blocking antibodies direct to ICAM-1 or with HA, as described above. The absorption values were determined at 405 nm wave length by Biorad spectrophotometer.

IEC apoptosis was evaluated by TUNEL assay (ApopTag from Oncor, Gaithersburg, MD) on cells stimulated or not with different doses of EVs and with RNase pre-treated EVs (50 µg/ml) for 48 hrs. Briefly, IECs were subjected to TUNEL assay (terminal deoxynucleotidyltransferase (TdT)-mediated dUTP nick end labeling) (ApopTag, Oncor, Gaithersburg, MD) after starving for 12 hrs without FCS. IECs were stimulated or not with different doses of islet EVs (1, 5, 10, 25, 50 µg/ml) and with RNase pre-treated EVs (50 µg/ml) for 48 hours. Cells were fixed in 1% paraformaldehyde, post-fixed in pre-cooled ethanol-acetic acid 2∶1, incubated with TdT enzyme in a humidified chamber at 37°C for 1 hr and counterstained with antidigoxigenin-FITC antibody and with propidium iodide (1 µg/mL). In selected experiments, EVs (50 µg/ml) were pre-treated with blocking antibody direct to ICAM-1 or HA, as described above. Samples were analyzed under a fluorescence microscope and green-stained apoptotic cells were counted in 10 non-consecutive microscopic fields.

IEC migration was evaluated by 10× phase-contrast objective under the above-mentioned Nikon system. Briefly, IECs were plated and rested for 12 hrs with RPMI without serum and subsequently stimulated or not with 50 µg/ml of Islet EVs, with RNase-pretreated islet EV or cultured in endothelial growth factor enriched medium (EBM medium, Lonza) for 6 hours. The net migratory speed (velocity straight line) was calculated by the MicroImage software based on the straight line distance between the starting and ending points divided by the time of observation. Migration of at least 30 cells for each experimental point was analysed [20].

Angiogenesis *in vitro* was evaluated as capillary-like structures formed by IECs (5×10^4^ cells/well) seeded on growth factor–reduced Matrigel (Beckton Dickinson, Franklin Lakes, NJ) in RPMI without serum. Briefly, IECs were stimulated or not with 50 µg/ml of EVs, RNase pre-treated EVs or cultured in endothelial growth factor enriched medium (EBM medium from Lonza, Basel, Switzerland) for 12 and 24 hrs. Cells were observed under a Nikon-inverted microscope, using a 50× magnification, and experimental results were recorded after 12 and 24 hours of incubation with different stimuli at 37°C. Results were given as average number of capillary-like structures/field (magnification×50) ± SD of three different experiments.

### Quantitative Real Time PCR

Quantitative real-time PCR was performed on total RNA extracted from islet EVs and IECs to confirm gene array data. Moreover, for selected experiments, the specific primers for human insulin were: F1-AAGAGGCCATCAAGCAGATCA and R1-CAGGAGGCGCATCCACA. First-strand cDNA was produced from total RNA using the High Capacity cDNA Reverse Transcription Kit (Applied Biosystems, Foster City, CA). Briefly, 200 to 400 ng mRNA, 2 µl RT buffer, 0.8 µl dNTP mixture, 2 µl RT random primers, 1 µl MultiScribe reverse transcriptase, and 4.2 µl nuclease-free water were used for each cDNA synthesis. After the reverse transcription, cDNA was stored at –20°C. 20 µl of RT-PCR mix, containing 1× SYBR GREEN PCR Master Mix (Applied Biosystems), 100 nM of each primer, and 0 µl, 1 µl, and 2 µl of MV cDNA, were assembled using a 48-well StepOne Real Time System (Applied Biosystems). Negative cDNA controls (no cDNA) were cycled in parallel with each run. Results are expressed as normalized values of mRNA mean differences (2∧^−ΔΔCt^ ± SD).

Quantitative real-time PCR for miRNAs was performed on total RNA extracted from IECs treated or not with islet-derived EVs and actinomycin D (Sigma). Briefly, IECs were treated with actinomycin D (5 µg/ml) for 30 minutes, in order to block RNA transcription, then washed and treated or not with EVs (50 µg/ml) at different time points. 200 ng of RNA was reverse-transcribed by the miScript Reverse Transcription Kit. cDNA was then used to detect and quantify miRNAs of interest by qRT-PCR using the miScript SYBR Green PCR Kit (Qiagen, Valencia, CA, USA). All samples were run in triplicate using 5 ng of cDNA for each reaction. miRNAs specific primers to hsa-miR-375, hsa-miR-200c and has-miR-21 were used in separate reactions. The snoRNAs, RNU48 and RNU44 were used as positive controls. qRT-PCR was performed using a 48- well StepOne Real Time System (Applied Biosystems). Negative cDNA controls (no cDNA) were cycled in parallel with each run. Results are expressed as normalized values of miRNA mean differences (2^∧−ΔΔCt^ ± SD).

### Statistical analysis

All data of different experimental procedures are expressed as average ± SD. Statistical analysis was performed by Student’s *t-*test or ANOVA with Newmann-Keuls or Dunnet’s multicomparison test where appropriated.

## Results

### Characterization of islet-derived EVs

**Figure 1:**
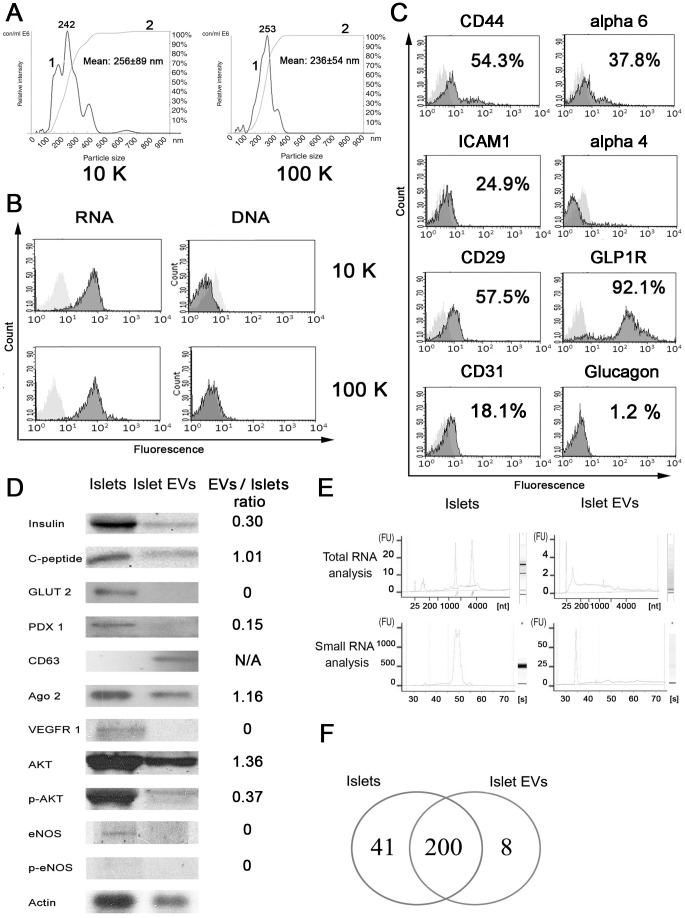
Characterization of human islet-derived EVs. A) Nanosight analysis of 10 k and 100 k fraction EVs. In Nanosight analysis, curve 1 describes the relationship between particle number distribution (left Y axis) and particle size (X axis); curve 2 describes the correlation between cumulative percentage distribution of particles (percentile in right Y axis) and particle size (X axis). B) GUAVA FACS analysis of 10 k and 100 k fraction EVs stained with acridine orange and evaluated for red fluorescence (RNA) or for green fluorescence (DNA). C) GUAVA FACS analysis of surface molecules (and glucagon) expressed by 100 k total EVs. Dark grey filled curves represent the percentage of positive cells in respect to control (light grey curves). Kolmogorov-Smirnov statistical analysis was performed (p<0.05). D) Western blot analysis of human islets and human islet-derived EVs for beta cell, endothelial cell and EV markers. Results are expressed as ratio of different protein levels between EVs and Islets normalized for beta actin ratio. Actin was used as experimental control. E) Bioanalyzer RNA profiling of human islets and islet-derived EVs for total RNAs (upper panels) and small RNAs (lower panels). F) Analysis of microRNAs (microRNA array) present in human islets and islet-derived EVs (left circle: islets; right circle: islet-derived EVs). Three different EV preparations were analyzed with similar results.

By Nanosight analysis, both 10 k and 100 k RCF (relative centrifugal force) EVs showed a similar size (256±89 nm and 236±54 nm, respectively), indicating that islet-derived EVs were only composed by small vesicles (Figure 1A). Larger vesicles, apoptotic bodies and cell debris were removed by 4000 g centrifugation. This was confirmed by GUAVA FACS analysis, showing that the 4 k fraction contained 10.3±2.7% of particles with a size >1 µm whereas large vesicles were absent in 10 k and 100 k fractions (not shown). The absence of apoptotic bodies in both 10 k and 100 k EV fractions was indicated by absence of DNA assessed by acridine orange test (GUAVA FACS) (Figure 1B). DNA was not detected in 10 k and 100 k EV fractions also after DNA extraction and analysis by agarose gel electrophoresis (not shown). We found that islets released 1.162±0.22 • 10∧6 EVs/IEQ. Moreover, 1 µg of EVs corresponded to 64.6±16.2 • 10∧6 EVs. GUAVA FACS analysis also revealed the presence on EV surface of several molecules such as alpha6- and beta1-integrin, CD44, ICAM-1, CD31 and GLP1R in both 10 k and 100 k fractions (Figure 1C). In EVs, glucagon was detected at very low level (Figure 1C). Moreover, the potential proliferative effect of the different EV subpopulations (10 k and 100 k RCF) was evaluated by BrdU assay on IECs and both subpopulations showed similar proliferative effect (not shown). Since the yield of vesicles was 100 times more in total 100 k fraction than in the 10 k fraction we performed further experiments with total 100 k fraction of EVs. Staining with PKH26 which labelled membrane phospholipids allowed detection of EVs by GUAVA FACS analysis indicating that particles detected were not protein aggregates. Western blot analysis of lysates derived from EVs showed the presence of insulin, C-peptide, the typical exosome marker CD63, the effector component of miRNA-mediated silencing complex Argonaute-2 (AGO-2) and molecules involved in cell proliferation such as AKT/p-AKT (Figure 1D). Furthermore, RNA extracted from islets and islet-derived EVs was submitted to Bioanalyzer profiling. In comparison to whole islets, EVs did not express the ribosomal RNA subunits 18S and 28S, whereas they were particularly enriched in small RNAs with a length of about 20–25 nucleotides, presumably miRNAs (Figure 1E). The comparative analysis of small RNAs showed enrichment in EVs (35±3.61%) in comparison to whole islets (17±2.82%). Moreover, EVs also contained RNAs of different lengths included between 40 and 400–500 nucleotides (Figure 1E). To further investigate the RNA content of islet-derived EVs, RT-PCR array for transcripts relevant to islet physiology was performed (table 1). EVs contained transcripts involved in different processes such as endothelial cell activation and angiogenesis (endothelial nitric oxide synthase, eNOS vascular endothelial growth factor alpha VEGFa), insulin production and signal transduction (PDX-1, insulin, insulin receptor, insulin receptor substrate2, PI3K subunits, AKT2), glycogen/lipid/protein synthesis (glucose transporter4, glucose-6-phosphatase, glycogen synthase kinase3) and peroxisome proliferator-activated receptor pathway (PPARA, PPARG, PPARGC1A, PPARGC1B) (Table 1). In particular, EVs were enriched for insulin mRNA and contained lower levels of glucagon and eNOS, suggesting their main beta-cell origin (Table 1). The presence in EVs of genes randomly chosen from those detected by array was confirmed by quantitative RT-PCR (not shown). We also performed TaqMan Array MicroRNA Card A, confirming the presence of several miRNAs within EVs as suggested by Bioanalyzer profiling. Indeed, we found 208 miRNAs within EVs (Figure 1F): 200 miRNAs were shared between islets and islet-derived EVs, whereas 8 miRNAs were specifically concentrated in EVs (Figure 1F and Table 2). By contrast, 41 miRNAs detected in islets were not present in EVs (Figure 1F). In particular, some shared miRNAs were enriched in EVs while other shared miRNAs were enriched in islets, suggesting a specific miRNA sorting mechanism during EV formation (Table 2). We found in EVs the presence of the islet specific miRNA miR-375 and of the so called “angiomiRs” which are known to promote angiogenesis (miR-126 and miR-296) (Table 2).

**Table 1:** Identification of mRNAs within islet-derived EVs by RT-PCR array.

Gene family	Gene	ΔCt	2^∧^-ΔCt	Gene family	Gene	ΔCt	2^∧^-ΔCt
**Receptors, transporters** **and channels**	ATP-binding cassette, sub-family C (CFTR/MRP), member 8	**2.7**	**0.153**	**Secreted factors**	Angiotensinogen (serpin peptidase inhibitor,clade A, member 8)	**3.2**	**0.108**
	Adrenergic, beta-3-, receptor	**4**	**0.062**		Chemokine (C-C motif) ligand 5	**3.2**	**0.108**
	Aquaporin 2 (collecting duct)	**3**	**0.125**		Glucagon	**0.6**	**0.659**
	Carcinoembryonic antigen-related cell adhesion molecule 1(biliary glycoprotein)	**3**	**0.125**		Interferon, gamma	**3.1**	**0.116**
	Glucagon-like peptide 1 receptor	**2.3**	**0.203**		Interleukin 6 (interferon, beta 2)	**3.7**	**0.076**
	Intercellular adhesion molecule 1	**2**	**0.25**		Interleukin 10	**2.7**	**0.153**
	Interleukin 4 receptor	**2.2**	**0.217**		Insulin	**-1.7**	**3.249**
	Insulin receptor	**3.6**	**0.082**		Resistin	**2.3**	**0.203**
	N-ethylmaleimide-sensitive factor	**2.2**	**0.217**		Tumor necrosis factor	**2.8**	**0.143**
	RAB4A, member RAS oncogene family	**2.7**	**0.153**		Vascular endothelial growth factor A	**3.4**	**0.094**
	Solute carrier family 2 (facilitated glucose transporter), member 4	**3.1**	**0.116**	**Signal transduction**	V-akt murine thymoma viral oncogene homolog 2	**3.9**	**0.066**
	Synaptosomal-associated protein, 23 kDa	**3**	**0.125**		Dual specificity phosphatase 4	**3.2**	**0.108**
	Synaptosomal-associated protein, 25 kDa	**3**	**0.125**		Inhibitor of kappa light polypeptide geneenhancer in B-cells, kinase beta	**3**	**0.125**
	Syntaxin 4	**2.5**	**0.176**		Inositol polyphosphate phosphatase-like 1	**3.3**	**0.101**
	Tumor necrosis factor receptor superfamily, member 1A	**3.4**	**0.094**		Insulin receptor substrate 2	**3.2**	**0.108**
	Vesicle-associated membrane protein 3 (cellubrevin)	**3.2**	**0.108**		Mitogen-activated protein kinase 8	**2.6**	**0.164**
	VAMP (vesicle-associated membrane protein)-associated protein A,33 kDa	**2**	**0.125**		Phosphoinositide-3-kinase, class 2, beta polypeptide	**2.6**	**0.164**
**Nuclear receptors**	Peroxisome proliferator-activated receptor alpha	**2.5**	**0.176**		Phosphoinositide-3-kinase, catalytic, delta polipeptide	**2.4**	**0.189**
	Peroxisome proliferator-activated receptor gamma	**4**	**0.062**		Phosphoinositide-3-kinase, regulatory subunit 1 (alpha)	**2.6**	**0.164**
**Metabolic enzymes**	ATP citrate lyase	**2.3**	**0.203**		Protein tyrosine phosphatase, non-receptor type 1	**3.8**	**0.071**
	Glucose-6-phosphatase, catalytic subunit	**3**	**0.125**		Tribbles homolog 3 (Drosophila)	**3.1**	**0.116**
	Glucokinase (hexokinase 4)	**2.3**	**0.203**	**Transcriptional factors**	CCAAT/enhancer binding protein (C/EBP), alpha	**3.5**	**0.088**
	Glycerol-3-phosphate dehydrogenase 1 (soluble)	**2.3**	**0.203**		Forkhead box C2 (MFH-1, mesenchyme forkhead 1)	**3.3**	**0.101**
	Glycogen synthase kinase 3 beta	**2.9**	**0.133**		Forkhead box G1	**2.7**	**0.153**
	Heme oxygenase (decycling) 1	**3.3**	**0.101**		Pancreatic and duodenal homeobox 1	**2.4**	**0.189**
	Insulin-degrading enzyme	**3.4**	**0.094**		Nuclear respiratory factor 1	**2.9**	**0.133**
	Malic enzyme 1, NADP(+)-dependent, cytosolic	**2.6**	**0.164**		Peroxisome proliferator-activated receptor gamma,coactivator 1 alpha	**3**	**0.125**
	Nitric oxide synthase 3 (endothelial cell)	**2.4**	**0.189**		Peroxisome proliferator-activated receptor gamma,coactivator 1 beta	**3.1**	**0.116**
	Poly (ADP-ribose) polymerase 1	**3.7**	**0.076**		Sterol regulatory element binding transcription factor 1	**3.9**	**0.066**
	Protein kinase, AMP-activated, alpha 1 catalytic subunit	**3.4**	**0.094**		HNF1 homeobox B	**2.5**	**0.176**
	Protein kinase C, beta	**3.2**	**0.108**		NK2 homeobox 1	**2.7**	**0.153**
	Phosphorylase, glycogen, liver	**3.3**	**0.101**				

Values are expressed as ΔCt (gene Ct – actin Ct) and as 2∧-ΔCt (gene Ct – actin Ct).

**Table 2:** microRNAs within islet-derived EVs by RT-PCR array and RQ values (Islet-derived EVs vs. islets).

miRNA	RQ	miRNA	RQ	miRNA	RQ	miRNA	RQ
hsa-let-7 a	0.612	hsa-miR-132	1.185	hsa-miR-223	28.219	hsa-miR-493	0.659
hsa-let-7 c	2.905	hsa-miR-133 a	7.641	hsa-miR-224	1.011	hsa-miR-494	1.200
hsa-let-7 d	0.931	hsa-miR-134	1.923	hsa-miR-296-5p	0.607	hsa-miR-495	0.866
hsa-let-7 e	0.926	hsa-miR-135 a	0.551	hsa-miR-301 a	0.561	hsa-miR-496	12.998
hsa-let-7 g	0.678	hsa-miR-135 b	0.384	hsa-miR-301 b	0.981	hsa-miR-500	1.523
hsa-miR-9	2.845	hsa-miR-137	2.465	hsa-miR-320	2.726	hsa-miR-502-3p	1.015
hsa-miR-10 a	12.575	hsa-miR-138	5.906	hsa-miR-323-3p	2.529	hsa-miR-505	1.757
hsa-miR-15 b	0.660	hsa-miR-139-5p	52.899	hsa-miR-324-3p	1.200	hsa-miR-517 a	18.619
hsa-miR-16	1.871	hsa-miR-140-3p	0.771	hsa-miR-324-5p	1.263	hsa-miR-532-3p	1.553
hsa-miR-17	0.006	hsa-miR-140-5p	1.185	hsa-miR-328	1.644	hsa-miR-532-5p	1.500
hsa-miR-18 a	0.333	hsa-miR-141	1.223	hsa-miR-329	1.683	hsa-miR-539	0.798
hsa-miR-19 a	1.021	hsa-rniR-142-3p	11.490	hsa-miR-330-3p	2.463	hsa-miR-545	1.137
hsa-miR-19 b	1.226	hsa-miR-143	4.566	hsa-miR-331-3p	0.716	hsa-miR-548a-3p	78.647
hsa-miR-20 a	1.113	hsa-miR-145	6.404	hsa-miR-335	3.096	hsa-miR-548c-5p	68.591
hsa-miR-20 b	1.088	hsa-miR-146 a	0.961	hsa-miR-337-5p	1.323	hsa-miR-551 b	1.489
hsa-miR-21	0.409	hsa-miR-146b-5p	3.130	hsa-miR-338-3p	2.863	hsa-miR-570	4.175
hsa-miR-24	0.499	hsa-miR-148 a	6.432	hsa-miR-339-3p	1.123	hsa-miR-574-3p	3.099
hsa-rniR-25	1.688	hsa-miR-148 b	2.700	hsa-miR-340	1.143	hsa-miR-576-3p	0.257
hsa-miR-26 a	1.669	hsa-miR-149	2.006	hsa-miR-155	2.597	hsa-miR-579	2.321
hsa-miR-26 b	1.598	hsa-miR-150	80.924	hsa-let-7b	2.474	hsa-miR-590-5p	1.845
hsa-miR-27 a	0.740	hsa-miR-152	5.370	hsa-miR-342-3p	2.172	hsa-miR-597	3.275
hsa-miR-27 b	1.472	hsa-miR-181 a	1.222	hsa-miR-345	0.954	hsa-miR-598	1.025
hsa-miR-28-3p	1.275	hsa-miR-181 c	2.046	hsa-miR-361-5p	1.494	hsa-miR-618	26.255
hsa-rniR-28-5p	0.723	hsa-miR-182	0.971	hsa-miR-362-3p	1.317	hsa-miR-625	2.743
hsa-miR-29 a	0.697	hsa-miR-183	0.402	hsa-miR-362-5p	0.853	hsa-miR-628-5p	2.101
hsa-miR-29 b	0.660	hsa-miR-184	0.191	hsa-miR-363	3.025	hsa-miR-629	5.911
hsa-miR-29 c	0.824	hsa-miR-185	0.736	hsa-miR-365	1.116	hsa-miR-636	7.712
hsa-miR-30 b	1.364	hsa-miR-186	6.007	hsa-miR-369-3p	1.377	hsa-miR-642	1.433
hsa-miR-30 c	1.987	hsa-miR-190	0.870	hsa-miR-372	2.454	hsa-miR-652	0.331
hsa-miR-31	0.982	hsa-miR-191	1.143	hsa-miR-374 a	0.927	hsa-miR-654-3p	0.485
hsa-miR-32	0.629	hsa-miR-192	1.630	hsa-miR-374 b	0.774	hsa-miR-655	0.775
hsa-miR-34 a	0.861	hsa-miR-193a-5p	0.972	hsa-miR-375	3.175	hsa-miR-660	1.960
hsa-miR-92 a	2.177	hsa-miR-193 b	2.231	hsa-miR-376 a	0.481	hsa-miR-671-3p	0.196
hsa-miR-93	0.900	hsa-miR-194	1.601	hsa-miR-381	2.222	hsa-miR-708	0.864
hsa-miR-95	1.212	hsa-miR-195	2.076	hsa-miR-383	1.221	hsa-miR-744	0.506
hsa-miR-96	0.498	hsa-miR-197	1.715	hsa-miR-410	1.922	hsa-miR-885-5p	2.857
hsa-miR-99 a	7.017	hsa-miR-199a-5p	1.761	hsa-miR-411	1.026	hsa-miR-886-3p	20.250
hsa-miR-99 b	1.958	hsa-miR-199a-3p	3.427	hsa-miR-422 a	19.012	hsa-miR-886-5p	22.840
hsa-miR-100	4.936	hsa-miR-200 a	1.132	hsa-miR-423-5p	1.610	hsa-miR-888	0.764
hsa-miR-101	2.082	hsa-miR-200 b	1.302	hsa-miR-424	8.294	hsa-miR-889	0.881
hsa-miR-103	0.729	hsa-miR-200 c	1.071	hsa-miR-429	1.064	hsa-miR-891 a	6.741
hsa-miR-106 a	1.121	hsa-miR-202	18.553	hsa-miR-451	5.904	hsa-miR-212	1.194
hsa-miR-106 b	1.295	hsa-miR-203	1.419	hsa-miR-452	0.992	hsa-miR-376 c	0.994
hsa-miR-125a-3p	0.646	hsa-miR-204	3.580	hsa-miR-454	0.947	hsa-miR-511	10.884
hsa-miR-125a-5p	0.633	hsa-miR-205	8.427	hsa-miR-455-3p	1.464	hsa-miR-133 b	N/A
hsa-miR-125 b	1.696	hsa-miR-214	7.543	hsa-miR-455-5p	1.356	hsa-miR-518 f	N/A
hsa-miR-126	14.684	hsa-miR-216 a	3.509	hsa-miR-483-5p	39.565	hsa-miR-519 d	N/A
hsa-miR-127-3p	1.441	hsa-miR-216 b	4.561	hsa-miR-484	2.39	hsa-miR-548 b	N/A
hsa-miR-128	1.355	hsa-miR-217	4.547	hsa-miR-485-3p	0.471	hsa-miR-548 c	N/A
hsa-miR-129-3p	1.209	hsa-miR-218	2.555	hsa-miR-487 b	2.019	hsa-miR-548 d	N/A
hsa-miR-130 a	1.357	hsa-miR-221	0.429	hsa-miR-489	3.222	hsa-miR-582-5p	N/A
hsa-miR-130 b	4.223	hsa-miR-222	1.287	hsa-miR-491-5p	1.670	hsa-miR-520 b	N/A

### Internalization of islet-derived EVs and induction of insulin mRNA expression within IECs

**Figure 2:**
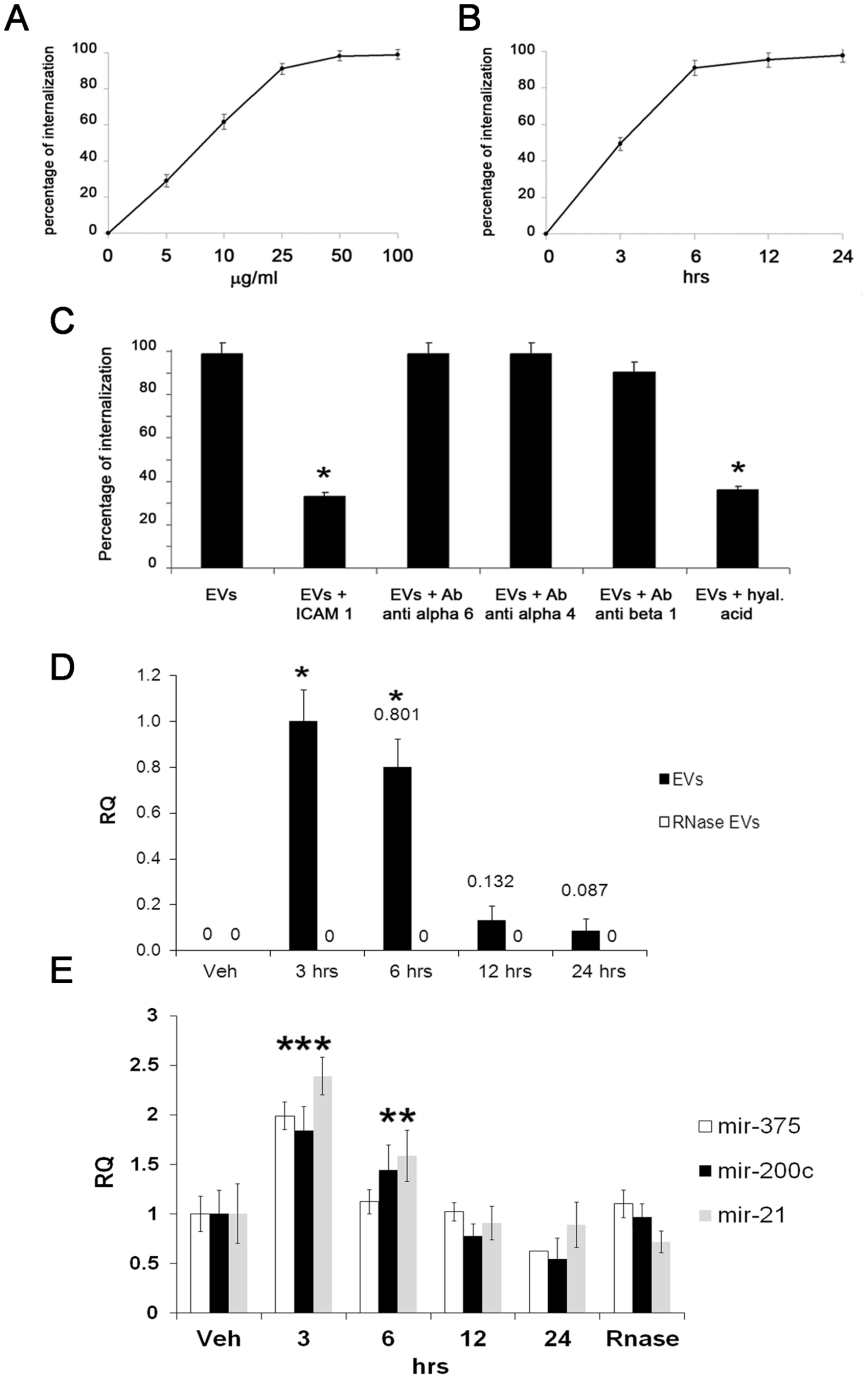
Internalization of islet-derived EVs into IECs and transfer of insulin mRNA and microRNAs. A–B) Quantification by GUAVA FACS of EV internalization in IECs at different doses (range 5–100 µg/ml) or at different time points with a single EV dose (50 µg/ml). C) Quantification by GUAVA FACS of internalization of EVs pre-labelled with PKH26 in presence or absence of hyaluronic acid or of blocking monoclonal antibodies directed to ICAM-1, integrins (alpha 4, alpha 6, beta 1) (*p<0.05 EVs+blocking monoclonal antibodies versus EVs alone). D) RT-PCR quantification of insulin mRNA in IECs treated with EVs at different time points. Results are expressed as normalized values of mRNA mean differences (2∧^−ΔΔCt^ ± SD) between each sample compared to IECs stimulated with EVs for 3 hrs (adopted as RQ = 1), (*p<0.05 EVs at 3 and 6 hrs vs. vehicle alone or EVs at 12 or 24 hrs). E) RT-PCR quantification of miR-375, miR-200c and miR-21 in IECs treated with actinomycin D (5 µg/ml) and EVs at different time points. Results are expressed as normalized values of miRNA mean differences (2∧^−ΔΔCt^ ± SD) between each sample compared to IECs treated only with actinomycin D (*p<0.05 EVs at 3 hrs or 6 hrs vs. vehicle alone). Three different experiments were performed with similar results. For FACS experiments, Kolmogorov-Smirnov statistical analysis was performed.

After labelling with the red fluorescent dye PKH26, we observed a dose-dependent internalization of islet-derived EVs into IECs (Figure 2A). EV internalization was already present 3 hrs after incubation reaching a peak at 12 hrs with a plateau at 24 hrs (Figure 2B). Experiments with blocking monoclonal antibodies were then performed to define the role of specific adhesion molecules expressed on EV surface in their internalization. By GUAVA FACS analysis, we found that EV internalization was significantly decreased in presence of a blocking antibody directed to ICAM-1 (Figure 2C). In addition, the inhibition of CD44 by incubation with HA significantly reduced EV internalization (Figure 2C).

We then investigated the potential transfer of mRNAs from EVs to target IECs. We selected as a marker insulin mRNA that was detected in islet-derived EVs. RT-PCR analysis demonstrated that IECs did not basally express insulin mRNA. However, the incubation with EVs induced in IECs the expression of insulin mRNA with a peak at 3 hrs and a subsequent decrease at 12–24 hrs (Figure 2D). EV-induced increase of insulin mRNA in IECs was not observed when EVs were pre-treated with RNase (Figure 2D). To further investigate EV-mediated RNA transfer, we analysed different miRNA expression in IECs treated with EVs and actinomycin D. Actinomycin D was used to inhibit RNA transcription in IECs in concomitance to EV stimulation. We selected miR-375, miR-200c and miR-21 as representative miRNAs since they were particularly enriched in islet-derived EVs. We observed that miR-375, miR-200c and miR-21 transfer was evident after 3 hrs of EV stimulation and subsequently decreased after 6 hrs (Fig. 2E).

### Anti-apoptotic and pro-angiogenic effects of EVs on IECs

**Figure 3:**
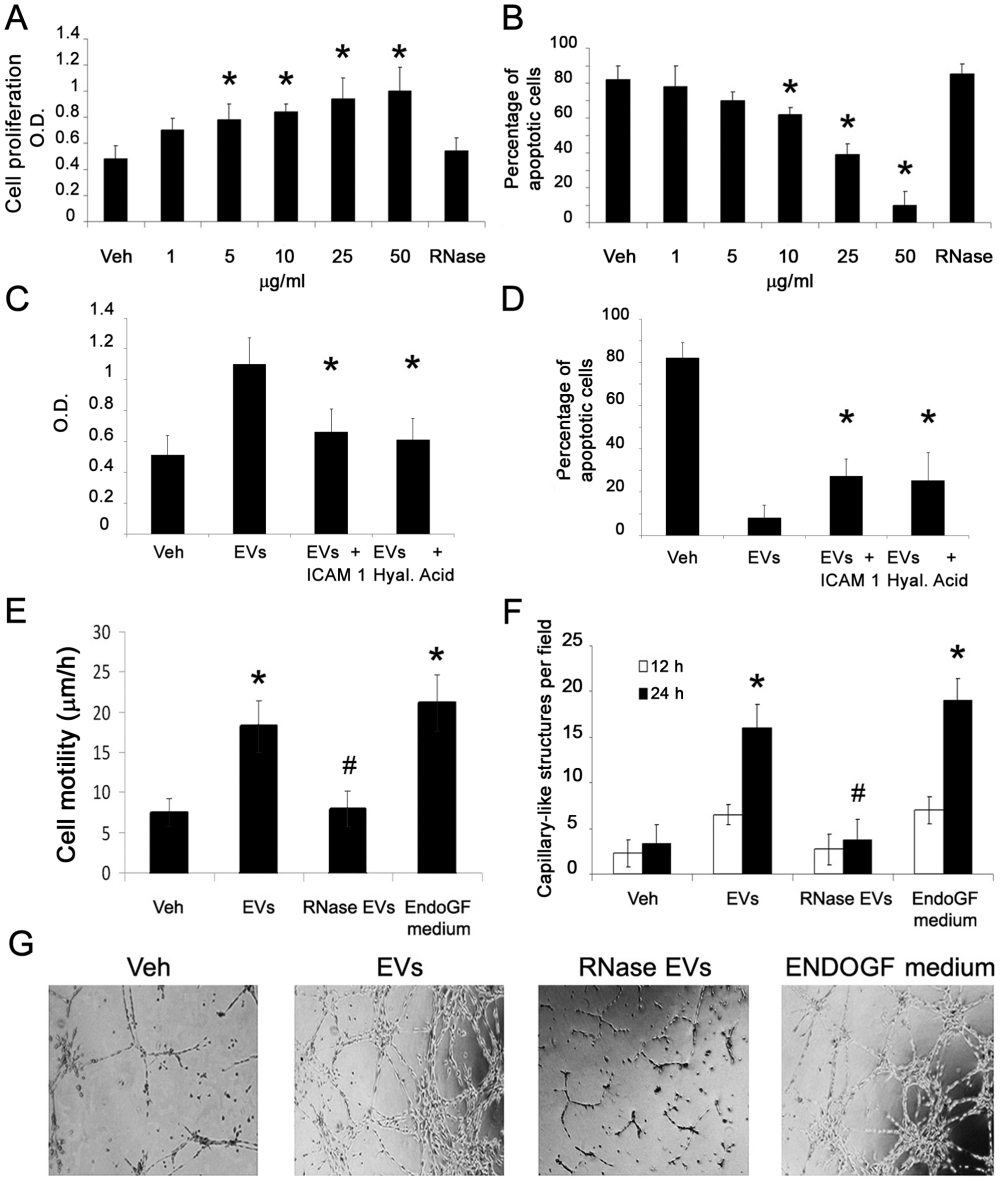
Pro-angiogenic and anti-apoptotic effects of EVs on IECs. A) BrdU proliferation assay of IECs incubated for 12 hrs with different doses of EVs or with RNAse pre-treated EVs (*p<0.05 EVs vs. vehicle alone or RNase EVs). B) Apoptosis (TUNEL assay) of IECs incubated for 48 hrs with different doses of EVs or with RNAse pre-treated EVs (*p<0.05 EVs vs. vehicle alone or RNase pre-treated EVs). C) BrdU proliferation assay of IECs incubated for 12 hrs with EVs (50 µg/ml) pre-treated or not with a blocking antibody directed to ICAM-1 or with HA (*p<0.05 EVs+blocking monoclonal antibody or HA versus EVs alone). D) Apoptosis (TUNEL assay) of IECs incubated for 48 hrs with EVs (50 µg/ml) pre-treated or not with a blocking antibody directed to ICAM-1 or with HA (*p<0.05 EVs+blocking monoclonal antibody or HA versus EVs alone). E) Time-lapse videomicroscopy analysis of 6 hrs IEC migration induced by EVs (50 µg/ml), EVs pre-treated with 1 U/ml RNase or by medium enriched for endothelial growth factors (EndoGF medium) (*p<0.05 EVs or EndoGF medium vs. vehicle alone; #p<0.05 RNase EVs vs. EVs). F–G) count and representative micrographs of *in vitro* formation of capillary-like structures by IECs seeded on Matrigel-coated surfaces in the presence of vehicle alone, EVs (50 µg/ml), RNase pre-treated EVs or medium enriched for endothelial growth factors (EndoGF medium) for 12 hrs (white columns) or 24 hrs (black columns) (*p<0.05 EVs or EndoGF medium vs. vehicle alone; #p<0.05 RNase EVs vs. EVs).

**Figure 4:**
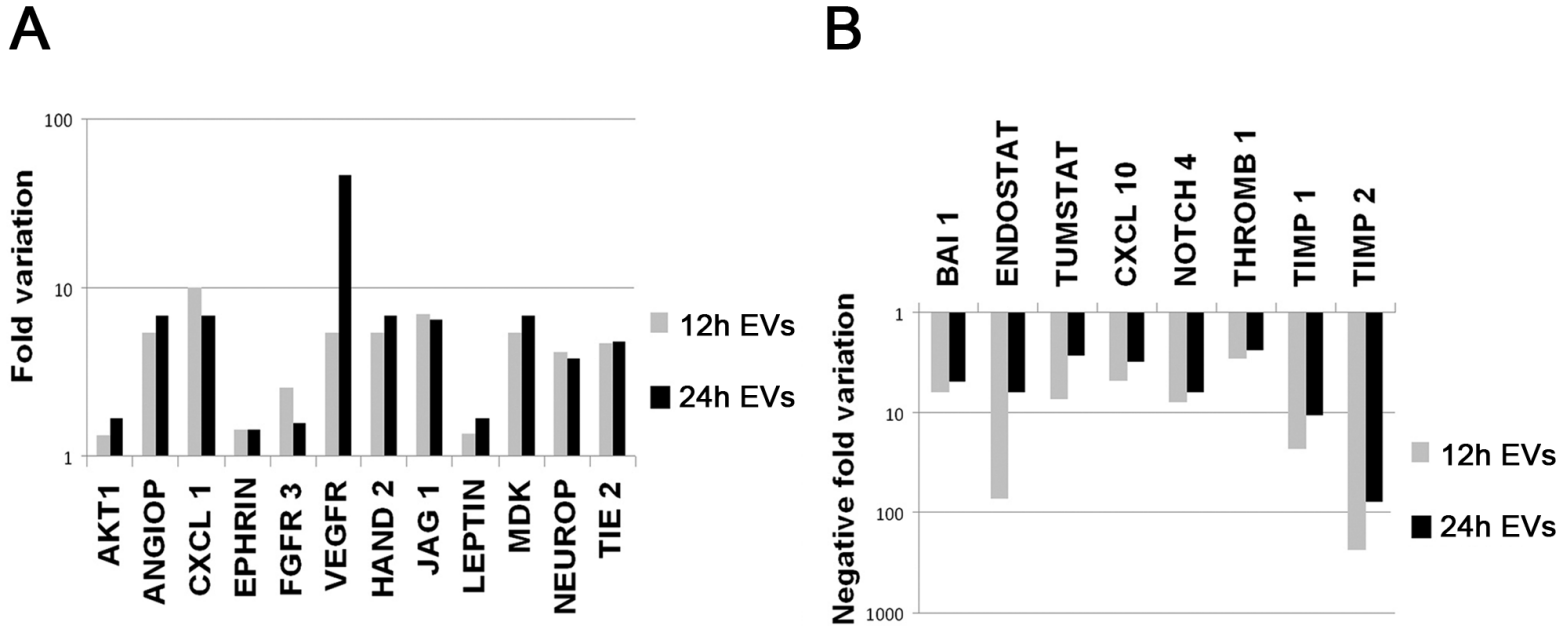
Representative gene array analysis for angiogenesis-related genes of IECs cultured in different experimental conditions. A–B) Results are expressed as fold-variation of pro-angiogenic and anti-angiogenic genes of IECs cultured for 12 hrs (gray columns) or 24 hrs (black columns) with EVs or with vehicle alone. Samples were normalized for signals generated by housekeeping genes (Actin, GAPDH). Three independent experiments were performed with similar results. Gene table: AKT1, Angipoietin1, CXCL 1, Ephrin A3, Fibroblast Growth Factor Receptor 3, Vascular Endothelial Growth Factor Receptor 1, HAND 2, Jagged 1, Leptin, MDK, Neuropilin 2, TIE 2, Angiopoietin 2, BAI 1, Endostatin, Tumstatin, CXCL 10, Notch 4, Thrombospondin 1, Tissue Inhibitor of metallo proteinases 1 and 2.

**Figure 5:**
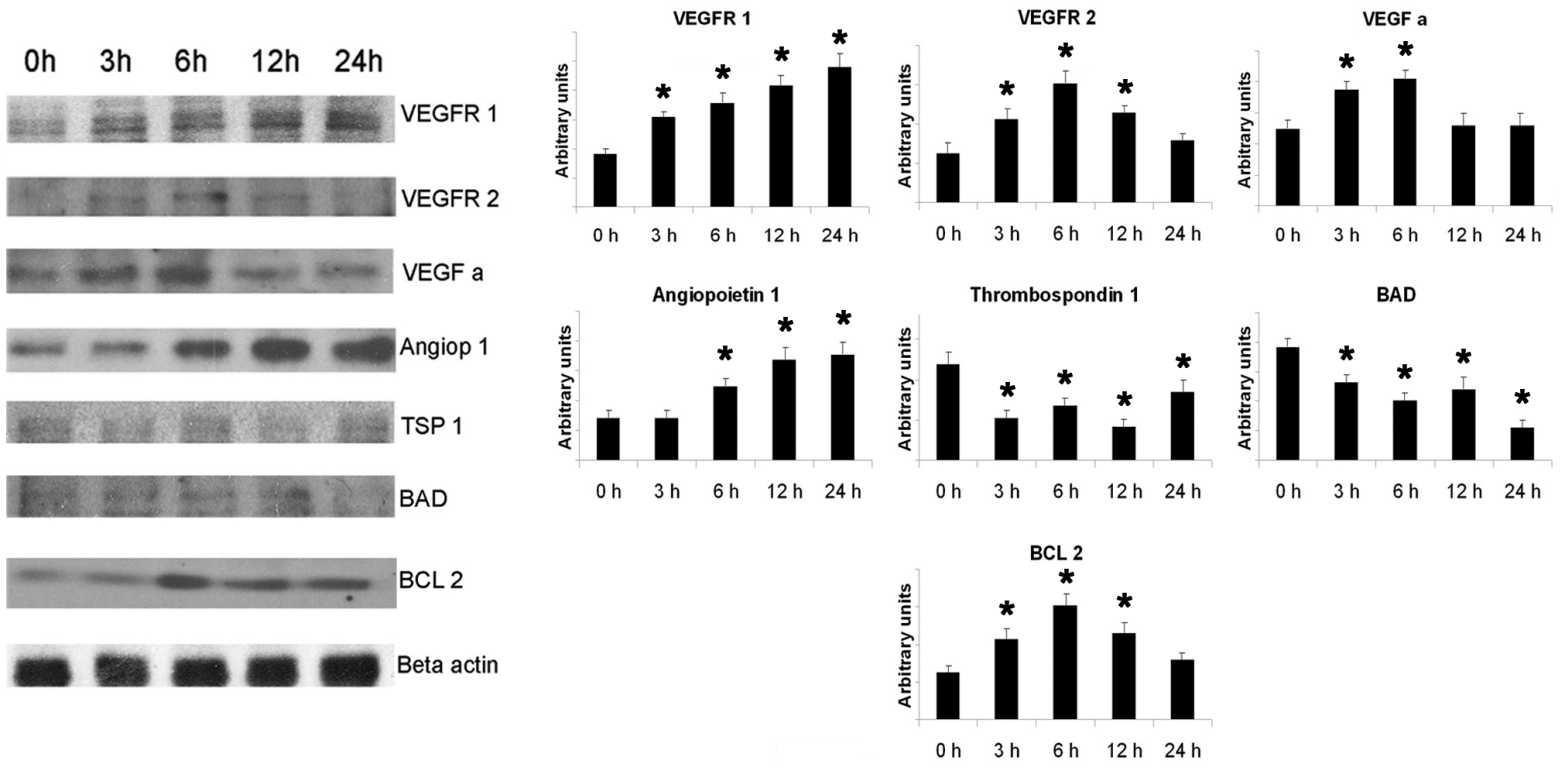
Representative western blot analysis and relative densitometric quantification of molecules involved in angiogenesis and apoptosis expressed by IECs after incubation with EVs at different time points. Results are expressed as ratio of different proteins (VEGFR1, VEGFR2, VEGF-A, Angiopoietin1, Thrombospondin1, BAD and Bcl2) in respect to beta actin (control). Three different experiments were performed and data are expressed as mean ± SD. (*p<0.05 EVs at different time points vs. vehicle alone).

**Figure 6:**
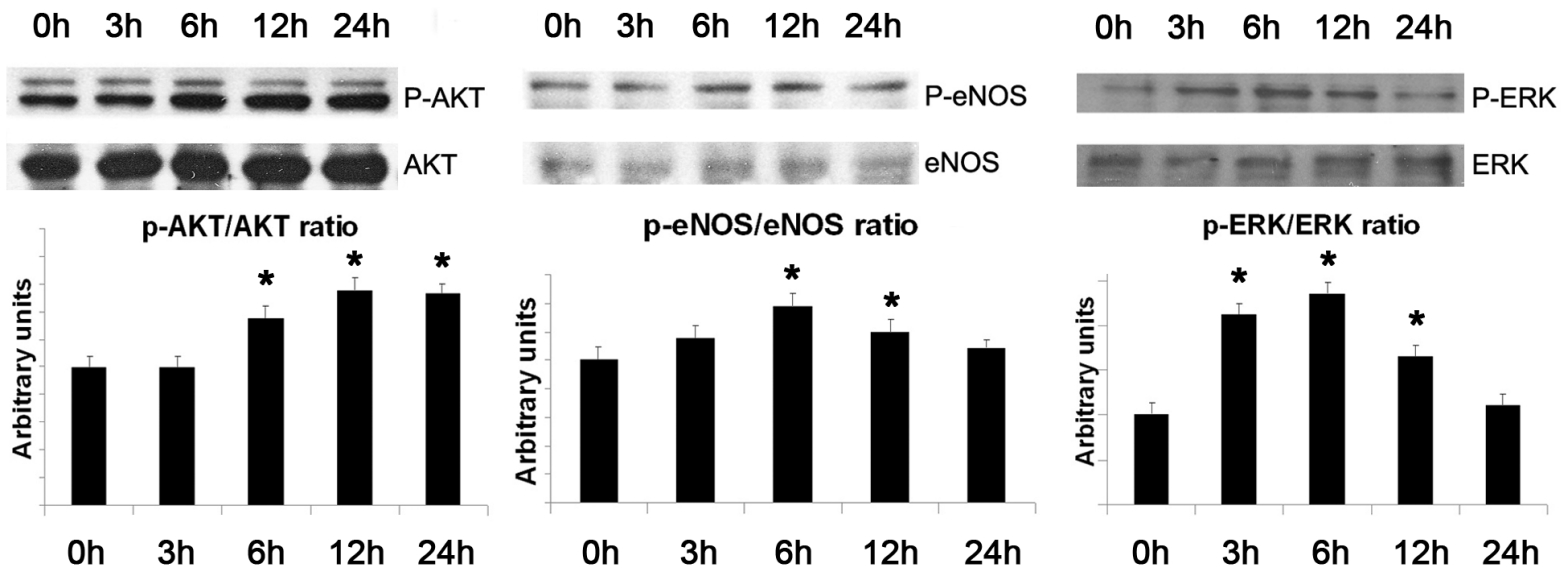
Representative western blot analysis and relative densitometric quantification of AKT, eNOS and ERK. Results are expressed as ratio between phosphorylated (P-AKT, P-eNOS, P-ERK) and non-phosphorylated (AKT, eNOS, ERK) forms. Three different experiments were performed and data are expressed as mean ± SD. (*p<0.05 EVs at different time points vs. vehicle alone).

As shown by BrdU assay, EVs induced a dose-dependent proliferation of IECs cultured in serum-free medium (Figure 3A). An inhibition of apoptosis of IEC after incubation with EVs was detected by TUNEL assay (Figure 3B). The proliferative and anti-apoptotic effect of EVs were significantly reduced when EVs were pre-treated with RNase, with a blocking antibody directed to ICAM-1 or with HA (Figure 3A–D). EVs also induced a significant increase of IEC migration (Figure 3E). Moreover, EV induced a significant increase of in vitro capillary-like structure formation after 12–24 hr incubation (Figure 3F and G). The migratory and pro-angiogenic activities of EVs on IECs were similar to stimulation with endothelial growth factor enriched medium (EndoGF medium). These effects were significantly reduced by RNase pre-treatment of EVs (Figure 3E–G). Gene array analysis revealed that EVs up-regulated IEC expression of several pro-angiogenic molecules such as angiopoietin1, TIE-2, VEGFR1, FGFR3, ephrinA3 and the endothelial transcription factor HAND2 (Figure 4A). Furthermore, EVs reduced the expression of anti-angiogenic factors including collagen fragments (endostatin and tumstatin), the adhesive glycoprotein thrombospondin1 and the tissue inhibitors of metalloproteinases TIMP1 and 2 (Figure 4B). These results were confirmed at protein level by western blot analysis. After EV stimulation, IECs showed an increased expression of several pro-angiogenic (angiopoietin1, VEGF-alpha, VEGFR1, VEGFR2) and anti-apoptotic factors (BCL-2) and the concomitant down-regulation of anti-angiogenic (thrombospondin1) and pro-apoptotic molecules (BAD) (Figure 5). In addition, EV stimulation induced the phosphorylation of intracellular signal transduction factors such as AKT, eNOS and ERK (Figure 6).

## Discussion

We herein demonstrated that freshly purified human pancreatic islets release biologically active EVs which may play a role in beta cell-endothelium cross-talk. The presence of proteins such as insulin, C-peptide and GLP1R and of high level of insulin mRNA within islet-derived EVs suggests that beta cells represent the main source of origin. Alpha cell and endothelial markers (such as CD31, eNOS and glucagon) were less expressed at both protein and mRNA level, suggesting that only a little amount of EVs did not derive from beta-cells, reflecting the normal architecture of cells within human islets. The internalization of EVs in IECs induced the expression of islet specific proteins and RNAs and reprogrammed IECs toward a pro-angiogenic and anti-apoptotic phenotype.

The presence of an intensive signal cross-talk between endothelium and endocrine cells inside pancreatic islets has been previously described [1]–[3]. Indeed, several experimental and human studies identified different factors able to mediate mutual signals in the adult pancreas [3]. Among these factors, the most studied was VEGF-A a pro-angiogenic growth factor secreted by differentiated endocrine cells. VEGF-A is able not only to attract endothelial cells and to induce angiogenesis, but also to stimulate formation of endothelial fenestrations essential for glucose sensing and interstitial flow. Moreover, other factors are known to contribute to survival and integrity of blood vessels such as the angiopietin-1 and the tyrosine-kinase Tie-2 ligand [22]–[24]. On the other hand, endothelial cells present in islets contribute to the production of extracellular matrix components involved in beta-cell physiology. In particular, proteins such as collagen IV, laminins and Connective Tissue Growth Factor are able to enhance insulin secretion, beta cell differentiation and specification of endocrine lineages. Moreover, HGF has been shown to induce beta cell proliferation in embryonic and postnatal pancreas [25]–[28]. It has been demonstrated that conditioned medium derived from cultured rat islets exerts a pro-angiogenic effect on islet-derived endothelial cells, confirming the presence of paracrine mediators involved in beta cell- endothelial cell cross-talk [9].

A growing number of evidences suggest that EVs may act as a new mechanism of cell-to-cell communication [10], [11]. Several studies described the involvement of EVs in diverse biologic processes such as tumor progression or tissue regeneration [29]–[33]. We have previously shown that EVs released from bone marrow-derived endothelial progenitor cells can be internalized by IECs and beta cells inhibiting apoptosis and promoting insulin secretion and angiogenesis in an experimental model of human islet xenotransplantation in SCID mice [14].

In the present study, EVs released by human pancreatic islets were shown to express the typical exosomal marker CD63 and different adhesion molecules such as alpha-6, beta-1 integrin, CD44 and ICAM1. Experiments using blocking antibodies demonstrated that ICAM-1 and CD44 are critical for EV internalization in IECs and consequently for EV-associated functional effects. EVs also shuttled typical beta cell marker such as insulin and C-peptide proteins, and AGO-2, a molecule required for miRNA-mediated silencing complex [34].

Several studies demonstrated that the biological effects of EVs from different origin can be ascribed to the horizontal transfer of genetic material ensuing in recipient cell reprogramming [13], [14], [35], [36]. The comparative analysis of miRNAs present within islets and those present in secreted EVs demonstrate an enrichment of certain miRNAs, suggesting that their compartmentalization within EVs is not a random process. Some of the miRNAs present in islet-derived EVs are known to modulate glucose homeostasis and to interfere with the typical complications of diabetes including nephropathy [37]. Of interest, EVs also carried a subclass of miRNAs called angiomiRs (miR-126, miR -296, miR -130 and miR -27b) which are known to possess a pro-angiogenic activity through the increased signaling of several growth factors (VEGF, EGF, FGF, PDGF) in endothelial cells [38]. Furthermore, RT-PCR array showed that islet-derived EVs contain different mRNAs of specific genes involved in islet physiology such as insulin, glucagon and PDX-1. Moreover, EVs carried also mRNAs of specific genes involved in endothelial cell biology and angiogenesis such as eNOS and VEGFa. The internalization of EVs in IECs was followed by the up-regulation of pro-angiogenic genes (angiopoietin 1, chemokine ligand 1, ephrin A3, FGFR3, VEGFR1, HAND 2, Jagged 1, Leptin, midkine, Neurophillin 2 and Tie 2) and down-regulation of genes involved in inhibition of angiogenesis (endostatin, tumstatin, thrombospondin 1, TIMP 1 and TIMP 2). The pro-angiogenic and anti-apoptotic effects of islet-derived EVs were confirmed at protein level by western blot analysis. EV stimulation induced the up-regulation of VEGFR 1, VEGFR 2, VEGF A, Angiopoietin 1, BCL 2 and the increase of the ratios p-AKT/AKT, p-eNOS/eNOS and p-ERK/ERK, pathways typically involved in endothelial cell activation. By contrast, thrombospondin 1 and BAD were down-regulated following EV stimulation. This resulted in an enhanced proliferation, an increased resistance to apoptosis and an in vitro angiogenic response of IECs. The relevance of mRNAs and miRNAs shuttled by EVs was suggested by the significant decrease of their biological effects on IECs when pre-treated with RNase. The insulin mRNA, which was absent in untreated IECs, was detectable at 3 and 6 hrs to decrease thereafter. RT-PCR analysis showed that EV treatment with RNase abrogated the expression of mRNA coding for insulin within IECs. In similar way, also miRNA-375, miRNA-200c, miRNA-21 were transferred by EVs in IECs at 3 hrs. These data suggest that islet-derived EVs shuttle several miRNAs and mRNAs which can play a role in beta cell-endothelium cross-talk.

In conclusion, human islets release biologically active EVs able to shuttle beta cell specific proteins, mRNAs and miRNAs. The incorporation of EVs in IECs is followed by a cell reprogramming toward a pro-angiogenic and anti-apoptotic cell phenotype. These results suggest a role of islet-derived EVs in the preservation of endothelial integrity and function.
